# Reconstruction With the Contralateral Fibula for Isolated Congenital Pseudarthrosis of the Fibula

**DOI:** 10.7759/cureus.100763

**Published:** 2026-01-04

**Authors:** Chikahisa Higuchi, Daisuke Tamura, Seiji Okada, Hidehiko Kawabata

**Affiliations:** 1 Department of Orthopedic Surgery, Osaka Women's and Children's Hospital, Izumi, Osaka, JPN; 2 Department of Orthopaedic Surgery, The University of Osaka Graduate School of Medicine Faculty of Medicine, Suita, JPN; 3 Department of Orthopaedic Surgery, Osaka Rehabilitation Hospital for Children, Osaka, JPN

**Keywords:** ankle valgus deformity, beta tricalcium phosphate, contralateral fibula transplantation, fibular osteosynthesis, isolated pseudarthrosis of the fibula

## Abstract

Congenital pseudarthrosis of the tibia (CPT) remains one of the most challenging conditions to manage in pediatric orthopedics. Fibular pseudarthrosis, often associated with CPT, is similarly resistant to treatment. In contrast, isolated congenital pseudarthrosis of the fibula (ICPF) is a rare condition for which no standardized treatment has been established. Here, we report four pediatric cases of ICPF treated with a novel reconstruction technique using the contralateral fibula. All patients achieved bony union at the pseudarthrotic lesion of the affected fibula. The donor sites of the contralateral fibula were reconstructed with β-tricalcium phosphate blocks. No fractures occurred at the reconstructed sites, and no recurrence of pseudarthrosis was observed. At final follow-up, which corresponded to skeletal maturity, all patients demonstrated mild ankle deformity on radiographs but no functional impairment.

## Introduction

Congenital pseudarthrosis of the fibula frequently coexists with congenital pseudarthrosis of the tibia. In contrast, isolated congenital pseudarthrosis of the fibula (ICPF) is seldom encountered in clinical practice. The incidence of ICPF remains unknown. To date, only 16 publications have reported on ICPF, comprising a total of 61 cases in the PubMed database [1-16]. On the other hand, no association between disease severity and patient age has been reported.

Dooley et al. proposed a classification system for ICPF based on fibular continuity and the presence of ankle deformities, with or without tibial involvement. In the more severe categories, grades 3 and 4, ankle valgus deformity develops as a consequence of fibular pseudarthrosis [1]. In general, the absence of fibular continuity can lead to progressive ankle valgus deformity during skeletal growth. Therefore, the primary goal of ICPF treatment is to prevent the progression of this deformity.

Here, we describe a novel osteosynthesis method for ICPF and retrospectively review four pediatric cases.

## Case presentation

Surgical procedure and cases 

**Figure 1:**
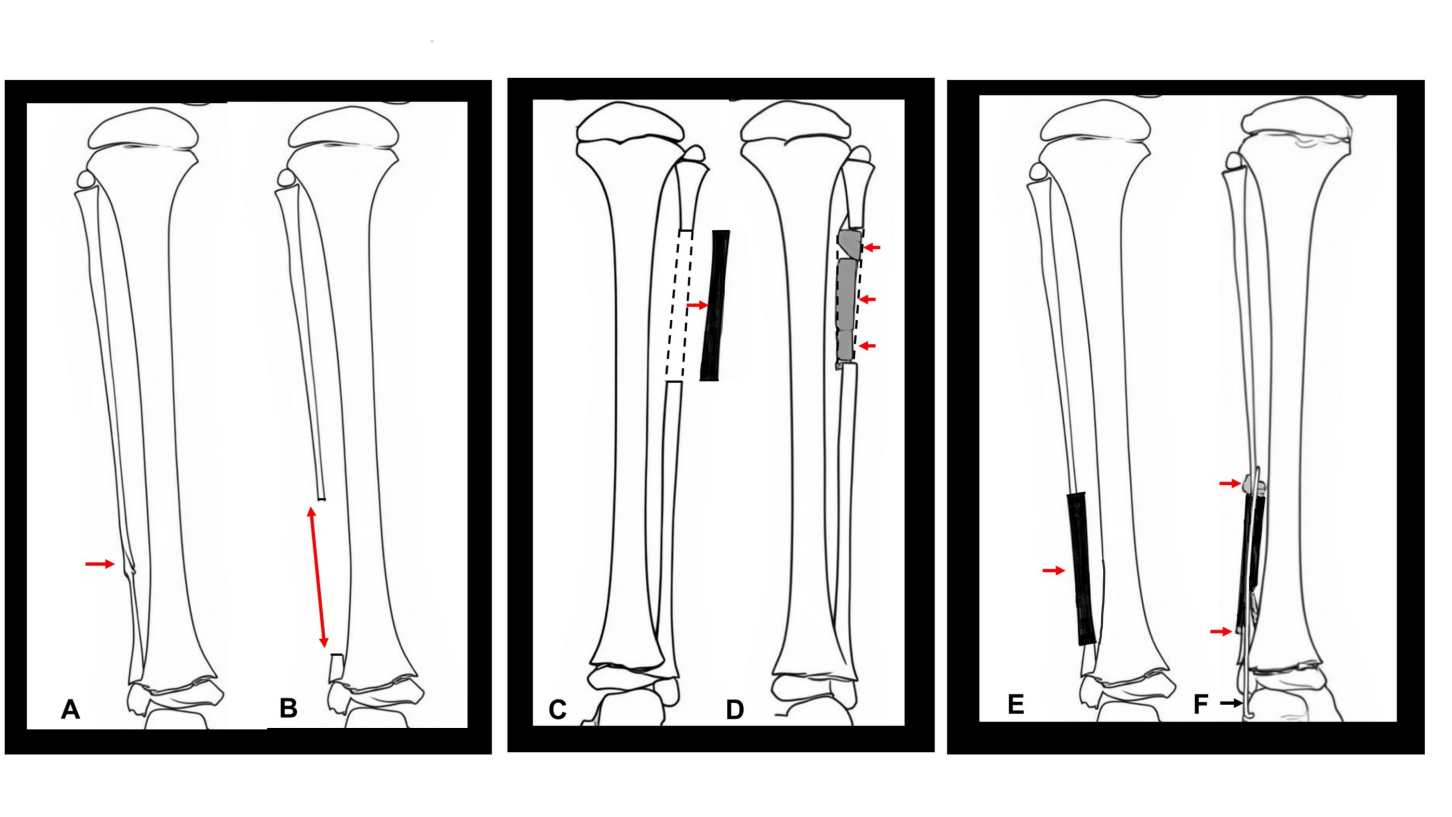
Diagrams of the surgical procedure (A) Pseudarthrosis of the affected fibula (red arrow). (B) Complete resection of the pseudarthrotic lesion with pathological periosteum (red double-headed arrow).  (C) A periosteum-free bone graft (black bar) was harvested from the contralateral fibula (red arrow). Dot lines show periosteum. (D) Beta-tricalcium phosphate blocks (dark gray bars) were inserted into its medullary cavity (red arrows) and then wrapped with periosteum (dot lines). (E) The harvested fibular graft (black bar) was transplanted interpositionally into the resected pseudarthrotic site (red arrow). (F) Kirschner wire was inserted for fixation (black arrow), and cancellous bone harvested from the iliac crest (light gray) was applied at the fibular junctions (red arrows), followed by coverage with a periosteal graft. Author's own work.

The osteosynthesis procedure for ICPF performed in this study is outlined below. First, the pseudarthrotic lesion, including the pathological periosteum, was completely resected from the affected fibula (Figure 1A, 1B). Second, a periosteum-free bone graft was harvested from the contralateral fibula (Figure 1C). Beta-tricalcium phosphate blocks were inserted into its medullary cavity and then wrapped with periosteum (Figure 1D). Third, the harvested fibular graft was transplanted interpositionally into the resected pseudarthrotic site (Figure 1E). Finally, cancellous bone and periosteum were harvested from the medial aspect of the iliac bone. Cancellous bone was applied at the fibular junctions and covered with a periosteal graft. Stabilization was achieved by inserting an intramedullary Kirschner wire into the intercalary fibular graft (Figure 1F). Postoperatively, a long leg cast was applied to the affected limb and a short leg cast to the contralateral leg until bone union was confirmed. After removal of the long leg cast, a lower-leg orthosis was used to prevent fractures of the reconstructed fibula.

Case 1

**Figure 2:**
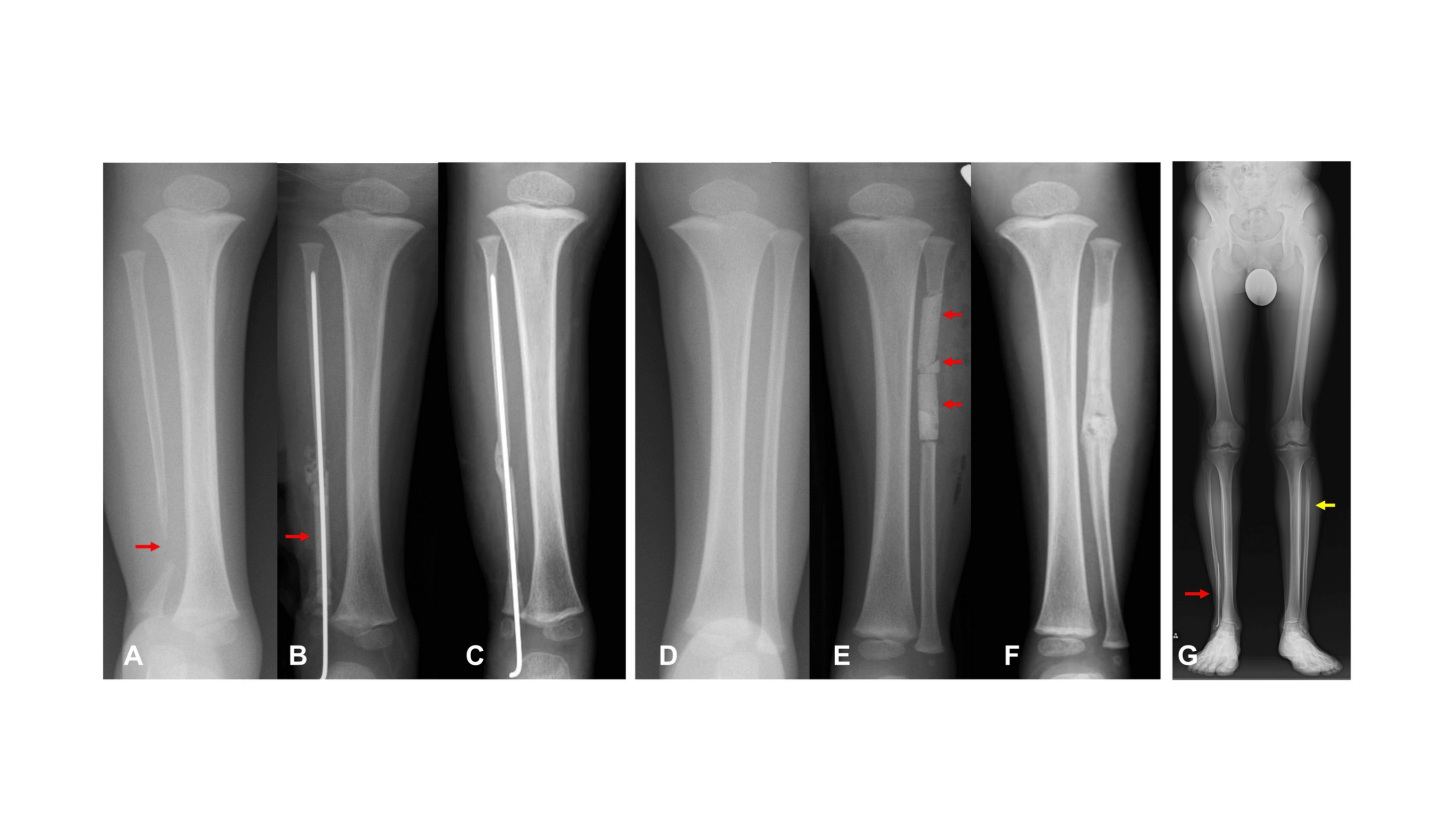
Case 1 (A) Pseudarthrosis of the affected fibula (red arrow). (B) Transplantation of the grafted bone (red arrow) with Kirschner wire fixation. (C) Bone union and restoration of fibular continuity. (D) The contralateral fibula before surgery. (E) Insertion of beta-tricalcium phosphate blocks (red arrows) into the contralateral fibula after graft bone harvest. (F) Bone union of the donor site of the contralateral fibula. (G) No recurrence of the affected fibula (red arrow) and Recovery of bony continuity in the contralateral fibula (yellow arrow).

A 2-year-old boy presented to the Department of Orthopedic Surgery at Osaka Women’s and Children’s Hospital (Osaka, Japan) with a chief complaint of right ankle deformity that appeared after he began walking. He was diagnosed with neurofibromatosis type 1. Radiographic examination at the initial visit revealed discontinuity of the right fibula, while the contralateral fibula appeared normal (Figures 2A, 2D). The condition was classified as Dooley type 3 isolated congenital pseudarthrosis of the fibula (ICPF), characterized by fibular pseudarthrosis without tibial involvement and accompanied by ankle deformity, as there was no history of fracture in the affected fibula. Surgery was performed at the age of 2 years and 1 month. Postoperative radiographs showed reconstruction of the affected fibula using a transplanted fibular strut and cancellous bone graft (Figure 2B), as well as insertion of beta-tricalcium phosphate blocks at the donor site in the contralateral fibula (Figure 2E). Bone union of the fibular pseudarthrosis was achieved 94 days postoperatively, and restoration of fibular continuity in the contralateral leg was observed 57 days after surgery (Figures 2C, 2F). At the final follow-up at the age of 17 years, there was no recurrence of fibular pseudarthrosis or discontinuity of the contralateral fibula. Radiographic evaluation revealed no recurrence of pseudarthrosis and complete regeneration of the contralateral fibula (Figure 2G).

Case 2

**Figure 3:**
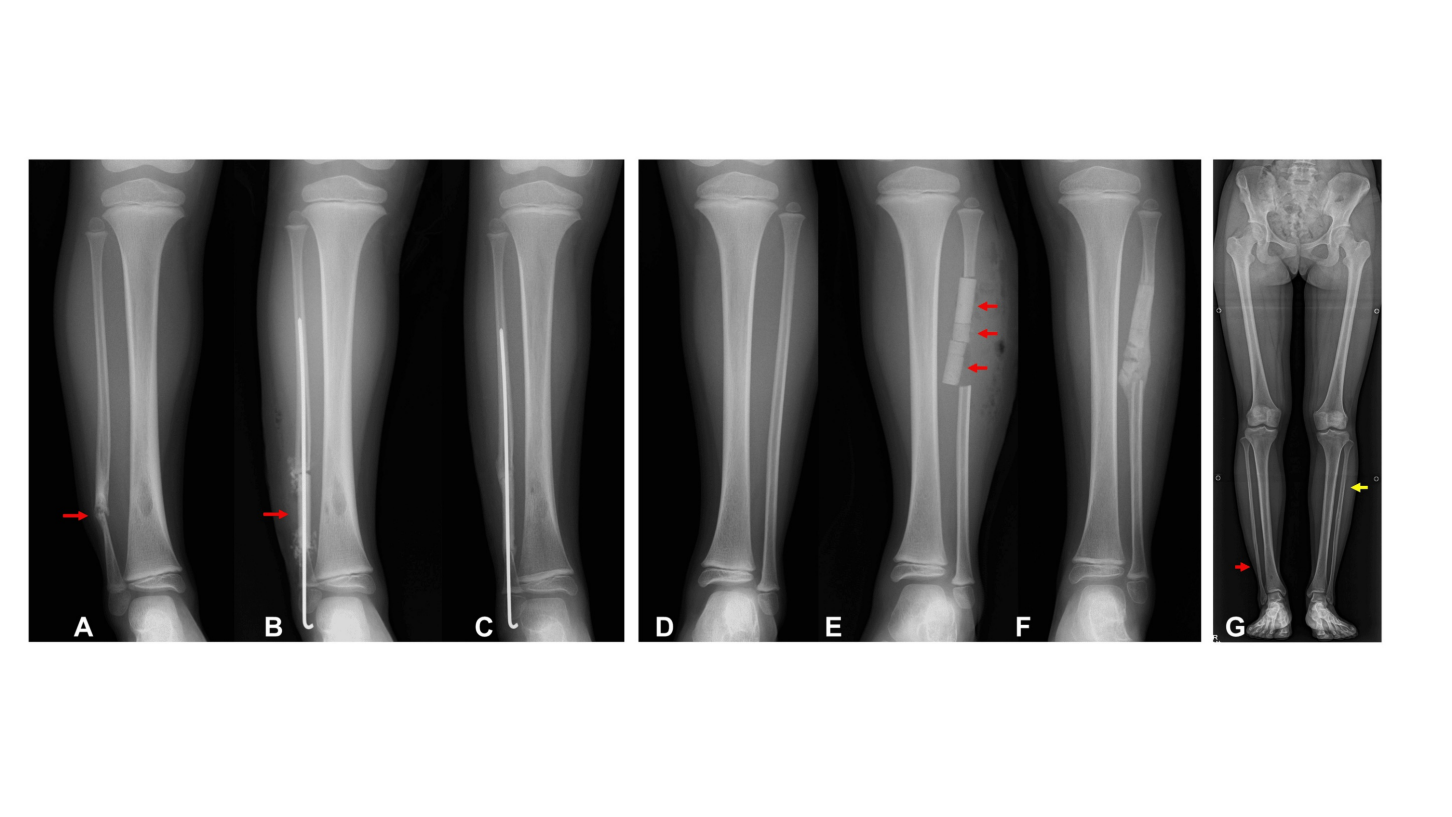
Case 2 (A) Pseudarthrosis of the affected fibula (red arrow). (B) Transplantation of the grafted bone (red arrow) with Kirschner wire fixation. (C) Bone union and restoration of fibular continuity. (D) The contralateral fibula before surgery. (E) Insertion of beta-tricalcium phosphate blocks (red arrows) into the contralateral fibula after graft bone harvest. (F) Bone union of the donor site of the contralateral fibula. (G) No recurrence of the affected fibula (red arrow) and Recovery of bony continuity in the contralateral fibula (yellow arrow).

A 4-year-and-3-month-old girl was referred to our hospital for evaluation and treatment of a right fibular deformity. She had been diagnosed with ICPF at a previous hospital. Radiographic examination at the initial visit revealed pseudarthrosis involving the distal one-fourth of the right fibula, accompanied by osteolytic changes in the right tibia (Figure 3A). Her condition was classified as Dooley type 2, characterized by fibular pseudarthrosis without associated tibial deformities. Radiographic evaluation confirmed that the contralateral fibula was normal (Figure 3D). Surgical reconstruction was performed when the patient was 4 years and 7 months old (Figures 3B, 3E). A biopsy of the tibial lesion obtained at the same time revealed fibrous dysplasia. Radiographic evaluation 69 days postoperatively demonstrated bone union at both the fibular pseudarthrosis site and the donor site in the contralateral fibula (Figures 3C, 3F). At the final follow-up, radiographs showed no recurrence of pseudarthrosis, mild varus deformity of the distal tibia, and complete regeneration of the contralateral fibula (Figure 3G).

Case 3

**Figure 4:**
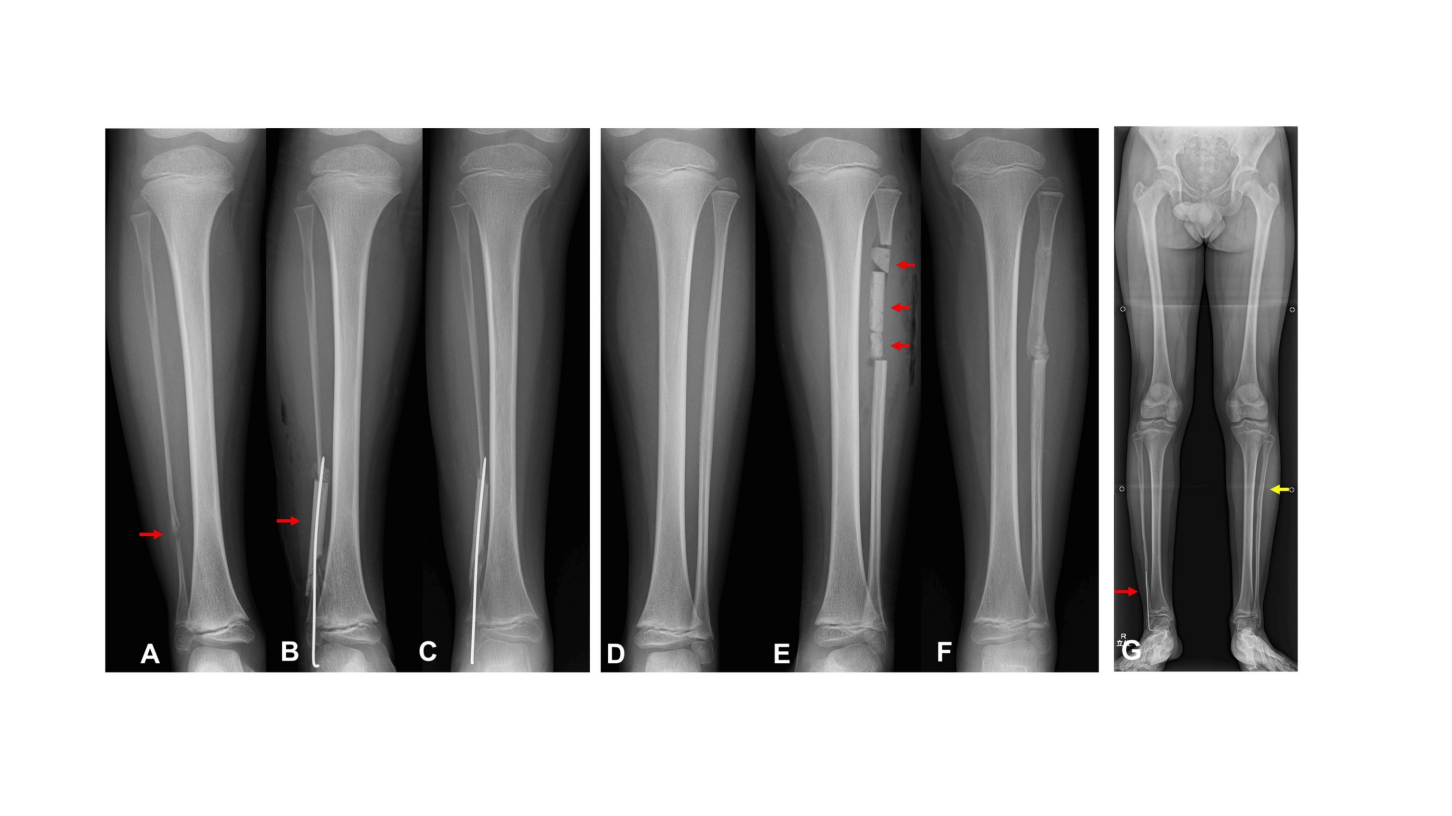
Case 3 (A) Pseudarthrosis of the affected fibula (red arrow). (B) Transplantation of the grafted bone (red arrow) with Kirschner wire fixation. (C) Bone union and restoration of fibular continuity. (D) The contralateral fibula before surgery. (E) Insertion of beta-tricalcium phosphate blocks (red arrows) into the contralateral fibula after graft bone harvest. (F) Bone union of the donor site of the contralateral fibula. (G) No recurrence of the affected fibula (red arrow) and Recovery of bony continuity in the contralateral fibula (yellow arrow).

A 7-year-and-2-month-old boy was referred to our hospital for the treatment of fibular pseudarthrosis (Figures 4A, 4D). He had previously been diagnosed with neurofibromatosis type 1 at another hospital, and we diagnosed him with Dooley type 2 ICPF associated with neurofibromatosis type 1. To prevent progression of ankle valgus deformity during the growth period, surgical reconstruction of the fibular pseudarthrosis was performed at the age of 7 years and 11 months (Figures 4B, 4E). Radiographic evaluation confirmed bone union at both the affected fibula and the donor site of the contralateral fibula 53 days after surgery (Figures 4C, 4F). At the final radiographic examination, performed at the age of 13 years and 4 months, no ankle deformity was observed in the affected leg, and remodeling of the donor site of the contralateral fibula was evident (Figure 4G).

Case 4

**Figure 5:**
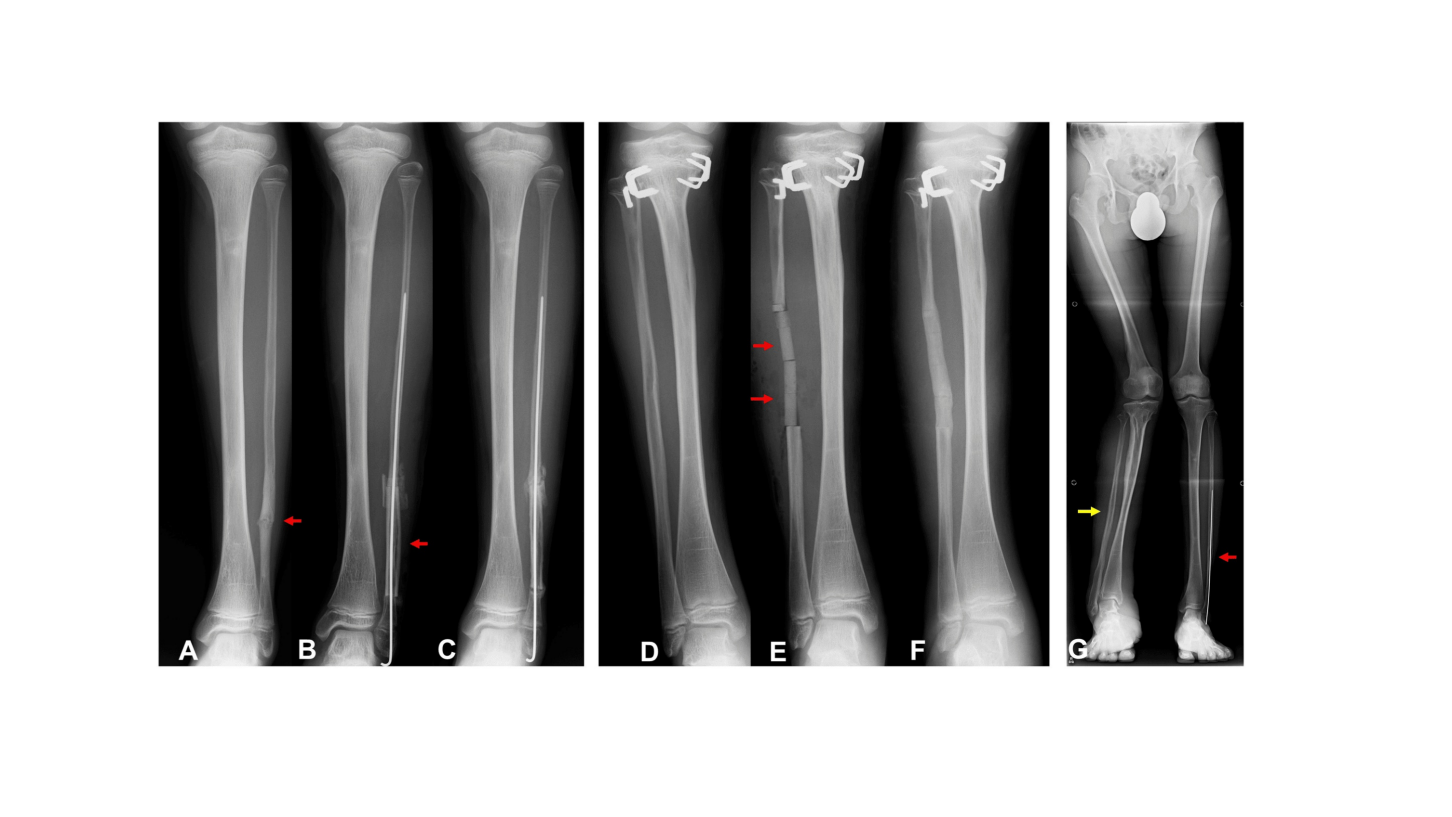
Case 4 (A) Pseudarthrosis of the affected fibula (red arrow). (B) Transplantation of the grafted bone (red arrow) with Kirschner wire fixation. (C) Bone union and restoration of fibular continuity. (D) The contralateral fibula before pseudarthrosis reconstruction surgery. (E) Insertion of beta-tricalcium phosphate blocks (red arrows) into the contralateral fibula after graft bone harvest. (F) Bone union of the donor site of the contralateral fibula. (G) No recurrence of the affected fibula (red arrow) and Recovery of bony continuity in the contralateral fibula (yellow arrow).

A 9-year-and-9-month-old boy was referred to our hospital with a fatigue fracture of the left fibula, which had been identified at a private clinic. He had previously been treated at our hospital for lower limb length discrepancy caused by hemihypertrophy of the right leg associated with neurofibromatosis type 1, at the age of 6 years and 5 months. Radiographic evaluation revealed pseudarthrosis of the left fibula (Figure 5A) and the contralateral leg that had undergone epiphysiodesis with staples (Figure 5D). He was diagnosed with Dooley type 2 isolated congenital pseudarthrosis of the fibula (ICPF), and the middle one-third of the right fibula was judged to have a normal structure. Reconstruction surgery of the left fibula was performed at the age of 10 years and 8 months (Figures 5B, 5E). Radiographic bone union of the left fibular pseudarthrosis was achieved 71 days after surgery (Figure 5C), while union of the donor fibula was confirmed 50 days postoperatively (Figure 5F). The final radiographic examination demonstrated continuity of both fibulas and a mild varus deformity of the left ankle (Figure 5G).

Summary of postoperative outcomes

No intraoperative or postoperative complications were observed. Radiographic union of the fibular pseudarthrosis was achieved in all cases, with a mean time to union of 71.8 days (range, 53-94 days). There were no instances of recurrence, graft fracture, or graft resorption during the follow-up period.

The mean time to radiographic union at the donor site of the contralateral fibula was 57.3 days (range, 50-69 days). No cases of pseudarthrosis or fracture of the contralateral fibula were observed during the follow-up period.

The lateral distal tibial angle (LDTA), defined as the angle formed by the tibial axis and the ankle joint line, was not directly affected by the surgical procedure, and no significant differences were observed between preoperative and immediate postoperative values. However, LDTA increased in all cases one year after surgery. During the follow-up period, three patients (Cases 2-4) demonstrated a progressive increase in LDTA. At the final evaluation, two patients (Cases 2 and 4) showed mild varus deformity of the ankle joint. In Case 1, the ankle valgus deformity that had initially improved one year postoperatively returned to its preoperative state by the final follow-up (Table 1).

**Table 1:** Change in LDTA of the affected and the contralateral sides Abbreviations: op: operation; M: month; Y: year; LDTA: Lateral distal tibial angle

Case	Before OP	After OP	6M after OP	1Y after OP	At final follow-up
1	81 (88)	81 (88)	81 (88)	85 (88)	81 (88)
2	87 (89)	87 (89)	89 (90)	90 (90)	97 (91)
3	83 (84)	83 (84)	83 (86)	88 (83)	90 (87)
4	90 (85)	90 (85)	91 (87)	93 (88)	94 (90)

## Discussion

ICPF is a rare disorder associated with various underlying conditions, and no standard treatment protocol has been established. According to case reports and review articles, management generally involves either distal tibiofibular synostosis or osteosynthesis of the pseudarthrosis. Several studies have investigated various therapeutic approaches for the osteosynthesis of fibular pseudarthrosis or distal tibiofibular synostosis. Some reports describe bone grafting procedures for fibular osteosynthesis, utilizing autologous bone struts from the iliac crest [2] or the induced membrane technique with iliac cancellous bone [3].

In 1967, Langenskiöld recommended distal tibiofibular synostosis as a treatment for progressive ankle valgus deformity associated with fibular pseudarthrosis [17]. This procedure involves resection of the pseudarthrotic lesion and achievement of bony union between the distal tibial and fibular metaphyses using an iliac bone graft. Distal tibiofibular synostosis has been considered an effective approach for stabilizing the ankle in ICPF. Dooley et al. also advocated for distal tibiofibular synostosis over osteosynthesis, citing the high risk of fibular nonunion, particularly in cases associated with neurofibromatosis type 1 (NF1), which closely resembles congenital pseudarthrosis of the tibia [2]. Yang et al. reported a case of ICPF in an NF1 patient who underwent this procedure at the age of three [4], and they recommended early distal tibiofibular fusion to prevent the progression of ankle valgus deformity. Dal Monte et al. described a case of NF1 in which grafted bone resorption occurred following an osteosynthetic procedure. Based on this experience, they advised fusion of the distal tibial and fibular metaphyses [5]. Similarly, Lampasi et al. suggested that distal tibiofibular fusion with autogenous bone grafting should be the preferred treatment for congenital pseudarthrosis of the fibula due to the challenges and complexity associated with fibular osteosynthesis [6].

However, this procedure presents certain challenges because it requires fusion of the distal tibiofibular joint. Although distal tibiofibular synostosis stabilizes the ankle, it also rigidly constrains the morphological contour of the ankle mortise. Under physiological conditions, the distal tibiofibular joint widens during ankle dorsiflexion to accommodate normal motion. Fusion of this joint, however, may alter the natural biomechanics of the ankle.

Frick et al. reported cases of children with iatrogenic synostosis of the distal tibia and fibula [18]. Their study highlighted that iatrogenic distal tibiofibular synostosis was associated with symptoms such as ankle deformity and pain. They suggested that such synostosis could restrict the normal distal movement of the fibula, impede ankle motion, and potentially lead to deformity or discomfort.

Given these considerations, distal tibiofibular synostosis should be reserved for select ICPF patients, such as adolescents, for whom its application may be more appropriate.

Several groups have attempted osteosynthesis for fibular pseudarthrosis. Traditionally, this approach involves complete resection of the pseudarthrotic lesion, including the pathological periosteum, followed by bone grafting from the iliac crest. Hsu et al. reported a case in which bone union of the pseudarthrosis was successfully achieved through bone grafting [7]. Similarly, Merkel et al. described a favorable outcome using iliac bone grafting [8].

Other groups have investigated alternative bone sources as substitutes for the iliac crest in the osteosynthesis of ICPF. In 1998, DiGiovanni et al. reported a case in which an allograft was used for fibular osteosynthesis [9]. In addition, Ng et al. applied the bone transport method using an Ilizarov external fixator [3].

Recently, novel techniques have been introduced to improve bone union in ICPF. Trigui et al. reported six cases of fibular discontinuity reconstruction using a periosteal flap technique without bone grafting [10]. They suggested that this approach is particularly suitable for young children. In 2017, Mansour et al. presented preliminary results of the induced membrane technique for ICPF, reporting successful bone union in all three patients at the final stage [11].

Several studies have compared fibular osteosynthesis and tibiofibular synostosis in ICPF. Cho et al. reported successful outcomes in four cases of fibular osteosynthesis using autografts and internal fixation. However, they also observed refractures in two of these cases. A review of these reports indicates that complications such as nonunion and refracture remain major challenges in osteosynthesis for pseudarthrosis and require careful management [1]. Martus et al. demonstrated that primary union was achieved through osteosynthesis with intercalary bone grafting in four of five ICPF patients, whereas synostosis failure occurred in two of three patients. Based on these findings, they recommended fibular osteosynthesis for ICPF, regardless of the presence of ankle valgus deformity, to restore fibular continuity. However, their study also noted refractures and early graft resorption, consistent with previous reports. In one case, revision surgery was performed using a rib autograft combined with bone morphogenetic protein. They further emphasized the importance of carefully maintaining ankle alignment in addition to fibular continuity [12].

Wang et al. reported 15 cases of congenital pseudarthrosis of the fibula (CPF) [13]. Their cohort included four cases of CPF without tibial involvement, seven cases of CPF with a tibial lesion but without pseudarthrosis, and four cases of CPF with tibial pseudarthrosis. Six patients underwent fibular osteosynthesis, and bone union was achieved in three patients with CPF without tibial involvement. In contrast, none of the cases with a tibial lesion achieved union. Based on these findings, the authors concluded that fibular osteosynthesis should be performed in cases of CPF without tibial involvement, a condition that closely resembles ICPF.

In this paper, we present a novel procedure utilizing the contralateral fibula to restore continuity in ICPF. Our method successfully prevented the progression of ankle valgus deformity, as demonstrated by changes in LDTA, and was associated with no cases of refracture or bone resorption. Furthermore, our approach offers two key advantages.

First, the grafted bone from the contralateral fibula can be precisely fitted to the defect following resection of the pseudarthrosis in both size and shape. In terms of donor bone size, the surgeon can readily obtain sufficient length to bridge the lesion and restore fibular continuity. Moreover, the tubular structure of the graft facilitates secure fixation between the native fibula and the graft using an intramedullary wire. Additionally, its circumferential cortical bone provides structural support, ensuring rigid fixation and reducing the risk of nonunion.

Second, our method results in less donor-site deformity compared to conventional procedures. The contralateral fibula, which serves as the primary graft source, can be reconstructed using beta-tricalcium phosphate blocks, allowing for subsequent regeneration. Moreover, iliac bone deformity is minimized because of the minimally invasive nature of our approach. Traditional iliac bone grafting often leads to significant deformity of the iliac crest, as both the lateral and medial cortices are removed, creating a large bone defect. In contrast, our procedure requires only cancellous bone and periosteum for transplantation, making it less invasive than conventional methods.

However, our novel technique also presents certain drawbacks. It involves three anatomical regions, like the affected fibula, the contralateral fibula, and the ilium, making it more invasive than traditional surgeries. In addition, treatment of the contralateral lower leg requires cast immobilization for several weeks, which may substantially affect patient mobility. These factors represent the principal limitations of our method. Such limitations may be addressed by modifications of our method, for example, covering the junction between the affected fibula and the graft bone with the normal periosteum of the affected fibula.

## Conclusions

We presented four cases of ICPF treated with reconstruction with the contralateral fibula. No recurrences and fractures at the pseudarthrotic site, and fibular continuity was maintained at skeletal maturity in all cases. In addition, none of the patients showed progression of ankle valgus deformity. Despite certain limitations, our reconstruction method may be recommended as a treatment option for ICPF.
